# Calibrating home, hospitality and reciprocity in migration

**DOI:** 10.1177/14634996221118140

**Published:** 2022-08-22

**Authors:** Nicholas DeMaria Harney, Paolo Boccagni

**Affiliations:** 6221 Western University, Canada; Università degli Studi di Trento, Italy

**Keywords:** Home, hospitality, reciprocity, migration and refugees, time–space

## Abstract

Hospitality, as an analytic and a lived experience, is central to the day-to-day workings of home, and to managing the tensions and contradictions inherent in place attachment and appropriation on any scale – from the domestic to the national one. This emerges as a contentious and yet under-researched social question whenever newcomers such as immigrants and refugees lay some claim for guesthood. Following this premise, and based also on our fieldwork, this article outlines a conceptual argument for a joint understanding of home and hospitality in time and space. This leads us to conceptualize ‘calibrated hospitality’ to appreciate the ongoing dialectic between the spatial, temporal, and relational dimensions of the host–guest encounter in immigrant- and refugee-receiving societies. Looking at immigrant and refugee inclusion in terms of hospitality being claimed, negotiated, and possibly denied, relative to the theories and practices of ‘home’, opens an extensive conceptual terrain for social research that is more connected to foundational lived cultural idioms, and contextually more sensitive, than approaches based only on policy frames such as integration, or on formal entitlements such as access or residence rights.

Home is the place where, when you have to go there,

They have to take you in.

Robert Frost – *The Death of the Hired Man*

## Introduction

The two lines in Robert Frost's poem capture the intimate connection between home, hospitality, time, and social relations – what we aim to systematically explore in this article, as an emerging heuristic for migration and refugee studies. Home is the critical site, and indeed *the* site, for interpreting hospitality since it locates in time and space the asymmetrical social relationships between host and guest that constitute hospitality. While always situated in space, a home is not a fixed place even if people constantly strive for control over some home-like structure in time (Blunt and Dowling, 2006; Brickell, 2012). Part of the imaginative connection to home is that it can provide comfort and security from uncertainties. Even so, it is a much more open, provisional and contested space. While home may sustain a sense of security and protection from some imagined and real outside that perceived solidity entails internal inequalities, assertions of authority, asymmetrical power relations, and potential or actual violence. Home is structured in a specific time through social relationships and encounters with people both inside and outside its physical and imaginative space, rather than being a place that is solely constituted internally by its members (Massey, 1994). Homes should not only be viewed simultaneously as structured in space and time, but also as unfixed and incomplete, since the social relations by which they are constituted are unsettled and shifting, thereby continually producing new social effects (Boccagni et al., 2020).

It is our argument that the phenomenological incompleteness of home is vividly illuminated by the experience, and by the analytics, of hospitality. Part of the ‘unfixity’ of home, and of its structuring from without as well as from within, has to do with the potential presence of guests – all those that, coming from outside, lay some claim for inclusion relative to those who are already in, or part of, home. As a result, hospitality, as a cultural idiom and a way to accommodate the claims of a potential guest, illuminates the shifting boundaries of home and, indirectly, its meanings and contents. The hospitable encounter challenges the complete hold of a place that a sovereign subject would call home (Yeğenoğlu, 2012). This is particularly critical, we argue, in everyday relations between ethnic or long-settled majorities and newcomer immigrants or refugees. Making sense of the social and cultural consequences of immigration has also to do with unveiling the deep-rooted and naturalized idioms of home and hospitality in each social and cultural context.

Drawing on our fieldwork both in migration and refugee studies, we first present the rationale for a joint understanding of home and hospitality, as co-constitutive of the lived encounter(s) between insiders and outsiders. We then show how home and hospitality are embedded in spatial and temporal dynamics that result in the negotiation of forms of *calibrated reciprocity*. This perspective can unveil everyday understandings of membership, more than an exclusive emphasis on formal entitlements or policy statements would allow us to do. To substantiate this, we explore processes of social and cultural distancing that reveal themselves through spatial and temporal registers. Finally, we revisit these distancing registers considering the social interdependence that is inherent in hospitality – the degrees of reciprocity that are calibrated by feelings of trust, intimacy, and familiarity as actors navigate these social worlds. Through this excavation of everyday reciprocity, within the spatio-temporal experiences of host–guest encounters, we illustrate the heuristic potential of the grounded entanglement of home and hospitality.^1^ This offers a contextually rich representation of everyday living with difference and a promising way to understand the social dynamics of coping with strangers, in the face of the sovereign desire to control a certain time–space. Throughout the article, we explore different manifestations and expected contents of ‘hosthood’ and ‘guesthood’. We do so from a broad positioning in so-called receiving societies, where any new arrival of a stranger as Simmel frames one – someone who arrives today and will stay tomorrow – bears an at least implicit claim for guesthood.

### Why home and hospitality: A conceptual overview

Hosting occurs in a ‘home’, whatever the scale, and concerns an outsider's claim to its resources in a particular time–space. It follows that hospitality as a cultural idiom and social practice reveals the tensions, social dynamics, naturalized assumptions, and structures that enforce social solidarity within that home. In short, it enables us to discern the expected reach, accessibility, and internal diversity of home. Part of hospitality's attraction as an analytic for thinking about ‘home’ is that it helps us consider the multiple layers of this concept, as a place, a metaphor, a set of relations and an aspiration (Boccagni et al., 2020). Both home and hospitality correspond to socio-spatial frames that can be scaled up to related forms of social solidarity, from the individual intersubjective encounter to different levels of identity – family, community, town, ethnic group, region, and nation. This scalar flexibility may obscure the differences between hosting in a home and other spaces. However, it also demonstrates the powerful effects of hospitality and home as metonymies for how people interpret inclusion and exclusion and evaluate the moral dimensions of their relations to other people in a contained space. This fluidity also speaks about how these concepts are densely entangled with time and space.

At the expanded scale of thinking of home and belonging, hospitality is a seemingly universally shared language to initiate exchange and interaction with strangers (Candea and Da Col, 2012; Kant, 1983 [1795]; Pitt-Rivers, 2012 [1977]). Each iteration of hospitality performs the negotiation of power, asymmetry, strangeness, and intimacy between host and guest in different ways, informed by different scales of space and time (Da Col, 2019; Herzfeld, 1987; Rosello, 2002). Derrida and Dufourmantelle (1997) focus on the idea of the ethical requirement of opening one's home to strangers as the primary ideal of hospitality, as opposed to a juridical one focused on rights. For Derrida hospitality's force and quandary is precisely the incompatibility and tension between the moral/ethical and the political even as they exist together awkwardly and chaotically. Unlike a focus on juridical rights, this leads us to focus on the nuance of human interaction and exchange in conditions of risk and uncertainty. To explore this interstitial space of social relations, we develop the concept of *calibrated reciprocity*, which enables us to conjoin the ethical/moral with the everyday assessments, tensions and speculations of the political/practical that must happen in these encounters. Increasingly, restrictive forms of control, detention and expulsion characterize one aspect of many state responses to ‘unwanted’ global migration over the past two decades. At the same time, states have adopted institutionalized forms of welcome to minimize the risks of uncertainty associated with the stranger-as-guest, that paradoxically are tied to procedural delays, welcome (detention) centres, off-shoring of processing, and time-limited humanitarian visas explicitly framed in the language of time-limited hospitality. In addition to these state and institutional actions, local forms of inclusion and exclusion and calibrations of intimacy provide a more complex picture of how migrants are differentially situated. Hospitality is then constituted by the tension between the ethical requirement to be open and welcoming to strangers and sovereignty's requirement to exclude them (Derrida and Dufourmantelle, 1997). In fact, hospitality is inherently contradictory in that hosts seek to manage, confine, and control the terms and conditions of their engagements. This desire to control and limit negates the very aspirational ethical and moral foundations of unconditional openness to strangers and difference.

The dynamism and flexibility of home and hospitality, as concepts, make them intriguing analytic terms to address the everyday encounter between settled, hegemonic, or long-term residents (an unstable, plural category) and the diversity of people on the move across internal and international borders. Yet, what deserves scrutiny is not just the specific way in which time and space are entwined and configure the connections between home and hospitality. To signal time and space as pivotal to such connections is also to call attention to proximity and distance as ways for people to assess social relationships and constitute cultural expectations. This leads us then to the dynamic third dimension of *calibrated reciprocity* that binds home and hospitality. In reciprocity, actors negotiate social relationships based on degrees of risk, (dis)trust, uncertainty, moral obligations, mutual recognition, sharing, and exchange that extend through space and time (Mauss, 1968 [1923–24]). A temporal framing structures reciprocity. It is guided by socially expected and sanctioned assumptions of the timing of how gifts received must be returned. While reciprocity can be balanced over time, as Mauss observed, hierarchy, inequality or asymmetry permeate our reciprocal relationships with the giver superior to the receiver. Even so, reciprocity refocuses our analysis from the self-interested individual to how culturally constructed social bonds, networks and collectivities are forged and form mutual obligations.

The work people do in reciprocity establishes, maintains, and controls obligations that are calibrated by their degrees of mutual trust and intimacy. *Calibration* suggests that this process is heavily dependent upon reading social settings and relationships for degrees of intimacy, assessment of risk, and social obligation. However, it also emphasizes that the negotiations of home and hospitality are active across plural territories, through multiple cultural registers and social domains. The relationships established through reciprocity are constant measures of the degrees of trust, obligation, and intimacy at play. In the case of home and hospitality, they potentially extend across multiple scales of differential solidarities, which include, for example, class, peoplehood, and neighbourhood. Herzfeld's (2005: 3) concept of ‘cultural intimacy’ identifies precisely how feelings of familiarity and closeness operate at scale to extend to groups as large as the nation. It is the ‘recognition of those aspects of a cultural identity that are considered a source of external embarrassment but that nevertheless provide insiders with their assurance of common sociality…’. That knowing nod of embarrassment marks the social contours of in-group belonging and acceptance. The sets of social relations mobilized in the making, maintaining, and imagining of a home and the performance of hospitality are characterized by calibrations of reciprocity. This depends on actual or anticipated social interdependence and degrees of previous familiarity that articulate the impression one would wish to produce on people.

In the sections that follow, we explore how these three elements – space, time, and *calibrated reciprocity* – illuminate our ways to interpret home and welcome others in it. In doing so, we draw from our ethnographic fieldwork with migrants and refugees in Italy. These are meant less to highlight distinctive research findings, than to provide fruitful resonances with the emerging literature on home, migration, and hospitality, and advance a more nuanced understanding of hospitality itself.

### On the spatial side of home and hospitality

Home and hospitality are united in their focus on a drive to assert sovereignty over a particular space through a mastery of time. As Pitt-Rivers (2012 [1977]: 514) noted, ‘A host is a host only on the territory over which on a particular occasion he claims authority’. This authority is an expression of sovereignty over a space called home. It is established by such things as memory, social relationships, kinship, property rights, ethnic or national group ideology, and length of stay. As many scholars emphasize, the meaning of home as a space of belonging also implies a social process of inclusion and exclusion (Ahmed et al., 2003; Boccagni, 2017; Brickell, 2012). Hospitality is a cultural idiom that provides a set of practices to lessen the perceived threat of difference with the arrival of a stranger in a place. The very arrival of an outsider threatens to destabilize the implicit rhythms, accommodations, synchronicity of actions, and the allocation of space and time that creates the implicit ‘commons’ in the home. These disruptions from ‘outside’ have the effect of revealing to members of the home the underlying spatial arrangements and settlements that operate in it. In this sense, the guest-as-stranger denaturalizes self-evident routines and mutual expectations within the home, regardless of their intentions (Schutz, 1944). A way to reduce the threat of the unknown or poorly understood outsider is to enmesh them in the mutual obligations of reciprocity that induces shared knowledge, experience, and trust and is the foundational dynamic of hospitality. This social device is deployed to sustain the current social order and assert control over a specific place, against the risk of uncertainty and the claims over that place that the stranger or guest might make.

The provisioning of food and other everyday resources is a central feature of planning (temporal) and spatial practice that ensures that all members of the home have what they need to sustain themselves (Douglas, 1991). What space is available for these common purposes, relative to a variety of individual needs and interests, is a mundane but critical question in everyday life at home – a part and parcel of the typically gendered work of home-making. Furthermore, that spatial allocation needs to be coordinated by members in the home to plan, distribute, consume, and restock those resources in space. As the members of the home are ordinarily on the move across space for work, school, or other reasons, the allocation and coordination of space must account for their fluctuating presence and its related provisioning when and where is necessary.

Against this backdrop, mobilities across international borders result in myriad forms of home-making that increase the complexity of the ‘where’ and ‘when’ of home (Cuba and Hummon, 1993; Massey, 1994). International migration reconfigures and recombines registers of hospitality in different home-like places where assertions of sovereignty and belonging are contested. Since ‘home’ is a situated, emotional, and imaginative space for migrants it may proliferate across different places through social relations that strive to retain it transnationally or to articulate it simultaneously in different locations (Harney, 1998; Levitt and Schiller, 2004; Tsuda, 2012). As they move or try to, across different places over time, migrants may develop numerous place-based attachments and elaborate social networks across different destinations (Fog-Olwig, 1997). In each of them, they may depend on (and sometimes demand) hospitable acts in other peoples’ places and homes. At the same time, most migrants do not disconnect from a previous home but maintain, develop, or reimagine home in the place they left through remittances, continual familial and social relations, or institutional networks (Boccagni, 2022; Harney, 2002; Marcelli and Lowell, 2005; Pierre-Louis, 2006).

With the disruptive entry of a guest, the pre-established settlements to divide the space of the home and sustain the needs of its members require recalibration both in a specific dwelling and also in the scaled-up metaphorical use of the nation-state. Who sits where at dinner? Which groups receive more cultural and political focus and resources? Who sleeps where? How are neighbourhoods and attendant services demarcated by racialized categories or recency of arrival? What forms of (cultural) interpersonal intimacy must be accommodated in space, etc.? Through this rupture, the naturalized or hidden domestic rules and inequalities surface. In that sense, strangers-as-guests are not just a threat from ‘the outside’ because of their presumed difference or even incommensurability to those assumed to belong in the emotional, social, and physical home. By their very existence, they also threaten to undermine the imagined internal solidarity and hegemonic naturalized authority of the home dynamics. This becomes particularly visible and problematic, whenever the imaginative, emotional, and moral repertoire of home reaches up on a national scale – the nation as home (Davies, 2014). On this scale, migrants can be framed as a threat to the body politic not only because of their foreignness, but also because their physical presence reveals the hegemonic, asymmetrical, and hierarchical interests that operate within the presumed horizontal solidarity of the nation (Anderson, 1991). Hospitality at this scale has a formal, reductionistic quality through state-sanctioned programs of welcoming, the moral posturing of the humanitarian discourse (Fassin, 2012), or the explicit use of a negative hospitality discourse to indicate an exhaustion with giving and sharing.

Indeed, the use of the idiom of hospitality in autochthons’ representations of migrants or refugees holds an interesting and paradoxical subtext. Migrants are precisely non-invited guests, as a central characteristic of the (labour) migrant paradox. This is something Zolberg (1987) captured with the famous phrase ‘wanted, but not welcome’. Refugees and related juridical categories are, in contrast, usually imagined to be temporary until the crisis that forced their migration is resolved. In practice, return to a ‘home’, though fundamental to the discourse of non-governmental organizations (NGOs), the UN and the people themselves, is practically, politically, and emotionally beyond attainment. Many refugees experience a sense of being stuck with a return to a version of home forever on the temporal horizon. On the face of it, calling someone a guest or the consistent use of the discourse of ‘welcoming’ (centres and programs) by state institutions is an act of acceptance and inclusion. It articulates the image of a home with open doors, unlike in the political imaginary of domopolitics (Walters, 2004). A case in point lies in domestic hospitality for refugees, as a constellation of citizen-led practices that undermine the everyday foundations of anti-immigrant discourses and politics (Boccagni and Giudici, 2021). Once we look at the public construction of migrants-as-guests more in fine grain however, we encounter an unexpected development. Hospitality, in certain terms, is less the reverse of domopolitics than a way to rephrase it. This is because the category of guest, by definition, has little to do with formal rights and duties. On the ideal-typical ends of a legal status spectrum, it can apply to an undocumented newcomer or a long-settled citizen whose ancestors arrived as immigrants and may face legacies of marginalization and racialization in a nation-state ethnic hierarchy.

Unlike more politically laden categories such as race, ethnicity, or religion, guesthood operates as a rigid boundary-marker between the in-group and the rest, but one can be simultaneously a guest and a host as one engages with different social relations and contexts. Relations, exchange, and interaction predicate its use. However, it may end up being equally essentialist, whenever it obliterates all other forms of external or internal categorization of guests themselves. On top of it, the benign tone that underpins the idiom of hospitality makes it all more difficult to unveil its exclusionary implications. It is probably not infrequent, for ethnographers of immigrant local integration, to encounter mundane and yet telling episodes such as the one Boccagni witnessed one Saturday night, 15 years ago, while doing fieldwork on the informal sociability of Ecuadorian immigrants in Italy. With a dozen more people, adults, and children, he was hanging towards the premises of a parish, where a new ‘Ecuadorian association’ was about to be founded. As a matter of fact, the local priest was acting as host for the nascent association. On the way to the building, Boccagni heard some screams that stood out from the already rich soundscape of those group hangouts. An Italian middle-aged man had suddenly popped up in the middle of the street, as an improvised football match among some children in the group had resulted in the ball bouncing on a car being parked right there. ‘Don’t forget you are guests here – you must behave as guests, you know what I mean?’, the man shouted across the group, encountering only a reaction of mild indifference. The entire scene lasted a couple of minutes, with no significant aftermath. Nevertheless, it stayed powerfully in Boccagni's memory. Although the complaints of that man were only a minor instance of everyday micro-conflicts in majority–minority relations, they were revealing something deeper and subtler than the ordinary ways to frame immigrants. While several of those present there were Italian citizens, or about to become such, this hardly affected the master way to frame them – people out of place, who could stay only on the terms set by pre-existing inhabitants. In short: guests.

For sure, this is not only the case for labour migrants in Italy. The narratives of Boccagni's Swedish–Somali informants in Rinkeby, a ‘super-diverse’ social housing district in outer Stockholm, were replete with equally essentialized ways to reproduce the host–guest divide. This operated easily, but not without consequences, in a discursive arena of formally equal (majority and minority) citizens. While the jargon, or even only the implicit idiom of hospitality did not slip into racist language, it effectively perpetuated the same foundational us–them separation. ‘They always see you like a guest – as if you don’t belong here’, said Ahmed, a Sweden-born young man of Somali descent. ‘How on earth could I feel home here?’ (Massa and Boccagni, 2021).

### On the temporal side of home and hospitality

To extend Massey's (1994) comments on the relationship between home, space, and time, the spatial dynamics of home and hospitality cannot be disentangled from the heterogeneous temporalities of migration (Axel, 1996; Cwerner, 2001; Fortier, 2006; Griffiths, 2014; Harney, 2020, 2014). The diverse temporalities of migrant trajectories, potentially marked by temporariness, transit, and uncertain duration of stay in a destination, threaten the control over space that is central to home-making and hospitable dynamics (Douglas, 1991; Miranda-Nieto et al., 2020). In this section, we explore how these heterogeneous and entangled temporalities disrupt the search for order in home and hospitality. We use a migrant narrative to demonstrate how the host–guest encounter occurs in different temporal frames and scales, which can generate uncertainty, misunderstanding, and tension.

Guests and hosts tend to have dissimilar perspectives on time. Each of them may be juggling perceptions, restrictions, responsibilities, and obligations in different temporal registers all at once. If the spatial qualities of home and hospitality are scalable, so are the temporal ones. The scalability of times of home and hospitality affects individual migrant experiences and collective narratives about how to integrate strangers through juridical categories, based on permitted durations of stay in a ‘sovereign’ territory. The coexistence between different registers of home and hospitality, and their mutual entanglements, complicate the social dynamics of host–guest relations.

As people organize provisioning and resources within a home, they think about needs – social and basic – across different durations. Members of a home draw on habit, experience, and memory to anticipate the present and future requirements and decide on the organization of their everyday social relationships to ensure daily life runs as smoothly as possible. The arrival of the (migrant) guest destabilizes that presumed order, which the host hopes to control by assuming a socially expected duration in the hospitable relationship (Merton, 1984). This typically amounts to a limited time frame, even if that duration is not specifically articulated but assumed. This finite duration is one measure of control the host can assert to ensure a manageable social encounter (Candea and Da Col, 2012; Derrida and Dufourmantelle, 1997; Herzfeld, 1987; Shryock, 2012). However, its potential indeterminateness, wherever there is no explicit arrangement in place, is part of the ambivalence of hospitality – and for that matter, of guesthood. Time threatens to undo the certainty hosts have over the control of space.

I arrived at Napoli Centrale, no place to sleep. I spoke no Italian and all the Africans that passed me were avoiding eye contact. Once they looked at me, they would have to take me home. On via Bologna, I spoke French to the Senegalese traders, and they set me up with a Burkinabé who took me into an apartment he shared with six others. It was hard for the first few weeks I slept in a bed, at first, in shifts, but gradually they let me carve out more space, a home. Abdoulaye (Burkina Faso, pseudonym)

Let us take the narrative of Abdoulaye, originally from Burkina Faso, whose migrant trajectory encompasses the heterogeneity of time, the various scales and multi-location of home, and the coexistence between different registers of hospitality. In 2008, Abdoulaye arrived at Rome's airport on a valid time-limited Schengen tourist visa that had been approved by the French Embassy in Burkina Faso, after French authorities acknowledged but ignored its validity. Suspecting that he might become a visa over-stayer, they had held him at Charles de Gaulle airport and forcibly sent him on a plane to Casablanca. According to Abdoulaye, as a school district head in Burkina Faso, he was forced to flee because of a conflict with a local Imam and was told by his superiors to get out of the country on owed-vacation time until things settled down. Since he was unable for personal safety reasons to return to Burkina, he bribed a gendarmerie in the Casablanca airport transit area, before passport control, to get a flight back to France. A flight to Rome was the only available option, which he took. Easily passing through Italian passport control, Abdoulaye approached an Italian police officer at Roma Termini train station and asked in broken French-English what to do. The response was, ‘go to Naples, that's home to Africans’. He arrived at the main Neapolitan train station on Piazza Garibaldi with no contacts and no place to sleep. He spoke no Italian and noted that all the Africans who passed him were avoiding eye contact. He felt they knew that once they looked at him, they would have to take him home – to show a stranger hospitality. On via Bologna, he spoke French to the Senegalese traders, and they set him up with a Burkinabé who took him into a shared apartment with six others. It was hard for the first few weeks, relying on the hospitality of co-nationals, but strangers. Abdoulaye slept in a bed, at first, in shifts, but gradually they let him carve out more of his own space. He relied on his hosts for everything at first but worked in the informal economy as a day labourer and carpenter to earn some cash, contributing to cooking, cleaning, and expenses so that he became part of the shifting constitution of the home. Moreover, his hosts introduced him to some nuns working with migrants. Through their help he obtained a temporary visa, which eventually after three 6-month renewals was turned into a 2-year renewable *permesso di soggiorno* (visa)*.* This finally allowed him the option to travel outside Italy with the right of re-entry. Meanwhile, he was in constant touch with his mother and friends in Burkina to check on them, send money back to support cousins in school and see if his political troubles may have subsided so he could return home. In time, Abdoulaye began to work for the Italian state refugee protection scheme (SPRAR), helping migrant minors settle. Recently, after his relocation to northern Italy to work with an NGO, Harney asked him if he does plan to return to Burkina permanently. He is not sure, especially not until he receives Italian citizenship. It is hard, by now, to know where he feels most at home.

The story of Abdoulaye's journey mixes multiple registers of hospitality, home, and time that often operate simultaneously. Simultaneity, we suggest, is unruly since it destabilizes clear and sequential connections between space and time in these interactions. First, he arrived with a state-sanctioned but short-term tourist visa organized through the French Embassy in Ouagadougou. Yet, in the event of travel and arrival, Abdoulaye's legal visitor-stranger status was denied in France but accepted in Italy. As a result, he was left in a suspended and precarious status once he realized he needed to apply for asylum since he had landed first in France in Europe, which according to the Dublin Regulation (2003) required him to submit an asylum claim in his first country of landing in the EU; but he was now in Italy and could not apply through its procedures as his second country of landing in the EU. In search of support, he arrived in Naples and depended on the hospitality of unknown but fellow, French-speaking West Africans, eventually co-nationals, and local Catholic nuns. His expectation, a future-oriented disposition, that there was a shared language of hospitality practised by ‘all Africans’ to one another, offered a sense of certainty even if it produced anxiety.^2^ Later, he gradually negotiated his status and acceptance by entering the rhythms and joint obligations of domestic space with fellow co-nationals. In short, this stranger became part of the home, and as a relational site, it demanded adaptation to its own temporal rhythms, while being affected by the external ones. Such fine-tuning in rhythms of time and space use is typically more complex under precarious legal conditions and in larger housing settings, including co-housing arrangements and collective facilities, in which migrants and refugees are over-represented (Miranda-Nieto et al., 2020). Over time, moreover, Abdoulaye's status as host has been extended into the extra-domestic sphere, as signalled by his role in the public schemes to receive precarious asylum seekers and refugees.

Throughout his migration experience, Abdoulaye encounters intersecting and heterogeneous homes and hospitalities and embodies different degrees of stranger-ness. Therefore, he must operate through different time frames of home and hospitality, with their attendant obligations and relationships. Engaged in the immediate hospitable relations in Naples, his future return to Burkina and obligations there affects his engagements in Naples and as a host for young asylum seekers. The temporality associated with hospitality informs his reconceptualization of home. What used to be home, that is, Burkina, becomes a subject of estrangement (Ahmed, 1999), which initially forces his migration. At the start of his trip, Abdoulaye seems to think he will return anyway, after a short stay in France. This, however, gets disrupted by his experience in Italy. His relationship to territory and home begins to transform over time such that he eventually becomes a host in the place where he was a guest, as an employee of an NGO that hosts young asylum seekers. Thus, the subject's transition from guest to host is a matter of legal status. It also emerges through a nuanced understanding of the diverse temporalities that impose serial time ‘limits’ on him, informed by changing legal status as much as by his own experiential journey and guest–host dynamics. Even when, at a later point, Abdoulaye does return to Burkina for holiday, feted by family and friends, he holds a status that combines host and guest. On the trip, he is accompanied by several Italian co-workers and friends, whom he wishes to host in his home country, while he also brings gifts for family members of those who had hosted him in Naples.

At a nation-state level, the complex entanglement of temporality and hospitality associated with the stranger is subject to institutional strategies of containment and simplification. Indeed, temporality is a key discursive frame that runs through popular, policy, and academic narratives about migration. To establish the stranger as ‘knowable’ through the ritualized social processes of welcome, the host tries to limit the degree to which the stranger shifts categories and, in doing so, potentially jeopardizes the assumed future sense of security of the national home and its members. Receiving states strive to control the strangers’ spatial and temporal experience as guests by setting legal status regimes that allocate them different kinds of permits and visas. Different socially expected durations are implicit in different policy frameworks, on a national and even on a local basis. These future-oriented policies calibrate, restrict, and transform the status of migrant ‘guests’ based on the supposed social, cultural, and economic effects of the duration of settlement. Nonetheless, the strategies deployed to cope with the stranger's categorical variation are unlikely to ‘freeze’ host–guest relations in a fixed and one-sided arrangement. They rather leave, by necessity, increasing scope for reciprocity. Abdoulaye's role in welcoming new migrant strangers to Italy is an instance of how the stranger's settlement may complicate the sameness assumed in nationalist ideology and the straightforward distinctions between host, guest, and entitlements to call a place home. Abdoulaye's work for non-profits as an intercultural mediator and later as a guide for young asylum seekers in a state program transforms his positioning from stranger–guest to a kind of adjunct welcoming host, part, but not fully of the Italian nation-state. He is tasked with explaining the codes of settlement for new migrants as an interpreter across cultural fields. As Harney was told by newer migrants, Abdoulaye had acquired special knowledge about Italians, Europe, the Italian state, and their future prospects.

### Calibrated reciprocity and social connectedness

Time and space open numerous sites and circumstances for joint consideration of the concepts of home and hospitality. Yet, what binds them socially and gives them meaning is the lived experience of the ways in which hosts and guests negotiate their respective obligations, familiarity, and intimacy, from asymmetrical positions of power and authority, by navigating shared knowledge in each space (Vigh, 2009). We frame this third analytical dimension as the process of *calibrated reciprocity.* People imagine, structure, and act in a ‘contained’ sovereign space to negotiate the asymmetrical engagements of hospitality through some form of reciprocity. The latter notion allows us to observe in nuanced ways the social and cultural dynamics of giving and receiving in homing and hospitable processes. These processes entail layers of social and moral obligations, connectedness, shared knowledge, and desires. They cannot be reduced to the motivations of a calculative individual (Mauss, 1968).

Abdoulaye’s story is a case in point. As he had trust in his knowledge of other unknown African migrants and assumed a shared language of hospitality with them, he was able to calibrate what social obligations he might request given the uncertain duration of his stay. Likewise, Abdoulaye uses a guest–host idiom in his counselling work with asylum seeker youth. He often reminds them of three entwined issues. First, they need to use their own cultural knowledge of African hospitality rituals and expectations to orient their interactions with the Italian state and non-profit workers who have provided them with sanctuary and opportunity. Second, as good guests, they need to take advantage of any opportunities provided to them, such as attending language and civics classes. Third, they need to be aware that this is not their country, and some Italians may be hostile to young African men. In that sense, Abdoulaye urged these migrants to calibrate proper social expectations and etiquette as guests in foreign space. This ‘etiquette’ manifested itself in numerous mundane, everyday ways about comportment on the bus such as properly validating tickets, sharing space, adjusting, or modulating conversational styles, tone and volume, showing courtesy to elderly travellers, etc. This advice was extended to not drinking beer in public spaces or how to share public parks.

At the same time, hosts, by activating the cultural idiom and social practice of hospitality, enact cultural expectations of sovereignty over an unruly space that they must negotiate with stranger-guests. Boundaries of social appropriateness are not fixed but enacted and tested as parties evaluate their degree of control over this space. The tensions and mediations inherent in sharing the same space are worked out through the idiom of hospitality. How is the (re)distribution of the resources of time, space, goods, attention, intimacy, etc., minimally shaped, or maximally put at risk? Moral judgements of equity, fairness, and degrees of intimacy between members inform the assemblage of affordances, cultural expectations, and certainties that constitutes the home. These forms of mutuality do not imply an absence of inequality, authority, or differential treatment within the home. They simply reveal that the home, of whatever scale, is a collective effort that demands sharing, sacrifice, and solidarity. The stranger or guest destabilizes that moral system and threatens the ability of members to anticipate the future based on these settlements. This requires a concerted social insistence to contain and assert sovereignty over the threat of a socially unruly space.

The guest's seeming acquiescence to the dominant rules in place is a critical aspect of this effort. As Boccagni's research in a refugee centre in Italy reveals, even the most mundane expectations on asylum seekers to comply with the norms for cleaning, entering and leaving the building have a moralizing dimension that is entwined with guest–host assumptions. These requirements are ‘for their own good’, for asylum seekers to get accustomed to autonomous housing and as an obligation given the fact that they are hosted for free. Scaling up from day-to-day interactions to the mainstream politics of refugee inclusion, a large critical debate has emerged on the fundamental ambiguity of refugee ‘activation’ (Brochmann and Hagelund, 2011; Kohl, 2020). The expectation that refugees on assistance engage in some form of active citizenship (e.g. volunteering, associational participation, etc.), while their own priority is typically to find whatever job soon, articulates a tension between multiple aims. This warns us to not assume that acquiescence or compliance is conformity. The host expectation addresses the need to enhance the guests’ human and social capital within this new unequal social context, and possibly to help them resist the narrative sense of passivity and emptiness associated with ‘waithood’ and past traumatic events (cf. Altin and Degli Uberti, 2022). However, it is also meant to encourage, or even enforce, a form of reciprocity. There is more than technical conditionality at stake here. More fundamentally, there is the expectation that refugees-as-guests demonstrate their gratitude (Healy, 2014) to (longer) settled groups-as-hosts, even while, strictly speaking, refugees have simply been exercising a human right under endangered life conditions. These calibrations are further complicated by the imperfect binary of guest and host as hosts are intersected with social inequalities, class, status, gender, etc. In a country with a considerable history of internal migration, frequent disparaging remarks by ‘hosts’ in northern regions towards southern Italians destabilizes the solidity of a host as does the different ways migrants are racialized and, in the process, situated in unequal positions. Formal rights are hardly a terrain that can fully satisfy the rationale and requisites of hospitality – whether from the point of view of hosts or, for that matter, of guests themselves. The terrain of hospitality is irremediably blurred, subject to ongoing negotiation, possibly open to contrasting feelings and understandings at the same time. In short, it calls for *calibrated* forms of reciprocity.

Boccagni's informant Olusola, like many residents in the same refugee centre, oscillated between uneasiness for his tough living conditions and uncertain prospects, and acknowledgement of the hospitality he had enjoyed. ‘Italy saved my life’, he told innumerable times. ‘I will ever be grateful for this hospitality … I came here naked, like a small child, and got the help I needed … but now I must make my own way and give back, by working and paying taxes, what I received’. The emphasis on the morality of reciprocity, in Olusola's narrative, was there regardless of any consideration of how ‘hospitable’ the local environment was, at the intersection between budgetary cuts on integration and the severe consequences of the pandemic on migrants’ already precarious employment. While complaining about structural racism around them, the young refugee informants repeatedly articulated a view of ‘Italy’ as a ‘host’ that had first rescued and then provided them with free housing and some economic assistance. This was a source of gratitude, albeit one that created uneasy dissonance with the backlashes to which Olusola and dozen thousands like him were exposed, as soon as their formal stay period was over, and they had to move and search for a bed place elsewhere. While vulnerability and the risk of further marginalization were part of their everyday lives, so was some concern about what we would call calibrated reciprocity – ways for the guest to assume the responsibilities, and prerogatives, of the host.

This is less a matter of grand reflections than of embodied, unreflexive everyday practice. It was part of Boccagni's staying in the refugee centre to have coffee with residents, whenever there was an opportunity for that – an obvious reminder of the critical role of conviviality under all circumstances, including those of (externally perceived) emergency (Vandevoordt, 2017). Having a coffee was an ordinary temporal marker in his visits to the centre, particularly with Said, a Pakistani in his late twenties. Buying coffees at the centre's automatic dispenser was expected to be a matter of taking and respecting turns, against Boccagni's unreflective inclination to buy coffee for Said all the time. The obvious differences in socio-economic position between the two were not expected to affect this coffee interaction – lest the interaction itself would become meaningless. Even such ordinary social practices reveal some scope for a guest-to-host transition although, of course, they do not affect the underlying power imbalances. In fact, the transition reveals coexistence between different registers of hospitality operating simultaneously – contrary to a binary view of hospitality and guesthood. People struggle to inform their proximate and everyday relations with some sense of reciprocity, because of social obligations and of their own sense of hospitality. This has implications in scaling and timing, including the ways in which guests approach hospitality – and possibly resist it – over time. In the instance above, Said's habit to buy coffee as much as Boccagni, or possibly more than him, can be read in multiple frames: not only as a form of silent resistance to the impotence of the state-sanctioned hospitality he is receiving, but also as a manifestation of an interpersonal desire for mutuality and obligation, and possibly as a habitual cultural idiom. While being ‘only’ a provisional guest in the centre, Said is also in the legitimate and valued position of a host – at least as far as buying a coffee goes.

Overall, migrants as guests unsettle the ordinary mutuality of the home on any scale, even if it is only for their external origin. To control the new dynamic threat of the stranger, the host uses established rituals of commensality, partitioning of the domestic space, and management for the duration of the visit. This varies and is calibrated along with the degree of intimacy the host feels for the outsider – guest, stranger, or foreigner – which also reflects racialized, gendered, and other categories of assessment. In the interpersonal domestic world of a home or a neighbourhood block, we can observe the small diacritics of trust and familiarity, values that undergird the feelings of intimacy. These reveal sets of interpersonal relationships which calibrate affective closeness by placing one's present and future at risk in the face of poor behaviour, dishonesty, or malfeasance of another. What is shared? Who does one rely on to watch children, support actions, store valued items, in times of need? On a larger scale, how does an imagined sense of intimacy inform preferential treatment of migrants from certain countries, over others? These are all empirical questions for ethnography, which can hardly be addressed unless on a context-dependent basis.

Hospitality draws on a moral economy of expectations and obligations that are less well-defined, and yet hardly less cogent than formal law provisions. The challenging question with respect to state-enacted hospitality is probably whether, if at all, it creates the conditions for hospitality to be returned or not – whether, and for whom, the roles of host and guest are amenable to be reversed. In contrast, the dense social obligations of home and hospitality in people's everyday negotiations reveal the limits of the juridical forms of exclusion and of policy prescriptions for inclusion. These rules and programs seek to provide clear processes of control, management, and decision-making over difference. Yet, they miss the spaces, time, and social dynamics of how life is lived. In fact, the everyday connectedness of calibrated reciprocity across inequalities in status, expected duration, and power offers a more profound indication of the capacity for living together in a spatio-temporal arrangement. What ‘calibrated’ reciprocity means in the migrant (or refugee) versus native (long-settled) interaction is then a matter of ongoing negotiation that is shaped, but not necessarily pre-determined, by large asymmetries in power, rights, and possibly in future life orientations between them.

## Conclusion

Thinking through home and hospitality forces us to consider the sharedness of a territorial space; that is, how people come together to share resources, resolve conflicts, and lay the groundwork for familiarity, if not trust. To that extent, home and hospitality return us to Douglas’ (1991) view of the home as a kind of commons in which the collective good, social obligations, and entanglements operate a degree of distributive justice. Home and hospitality both acknowledge the shared experiences, expectations, and future of host and guest towards a kind of existing or aspirational collectivity or assemblage that requires a focus on relationality. The notion of calibrated reciprocity helps us observe these processes in time and space. Calibrated reciprocity reveals the subtleties of host–guest relationships as enacted in specific time–spaces, and then as evolving over time, thereby advancing our understanding of two critical points. First, host and guest are not fixed or essentialized. They are mutually exclusive identities within specific relationships but the categories can also be contested and changed over time or situationally. They are relational and mutually oriented positions, even when their relationship is shaped by major inequalities between the relevant parties. Second, the extent and ways in which guests wish and can ‘reciprocate’ are not given in advance, nor subject to any formal rule. However, they are subject to relatively shared and consistent expectations that are calibrated in the lived experience of host–guest relations. As such, they call for ethnographic understanding, rather than resting only on abstract or normative accounts. As far as migrants and refugees are concerned, the capability and even as some might suggest, right to transition from guests to hosts, whenever one wishes to, could be seen as a meaningful focus for ‘integration’ – one no less valuable than ordinary indicators related to labour market participation, language acquisition, intermarriage, and thus forth.

In practice, reciprocity is probably bound to be partial – just like hospitality is bound in time and space, and home itself is ultimately incomplete. The incompleteness of home and the tensions inherent in the boundary-making between the seemingly fixed and the relational aspects of home, as illuminated by hospitality, are sharpened by the indeterminacies of migration. Home as imagined by states and media publics is a comfortable middle-class dwelling with ample space and well-stocked with provisions. This imagery leaves little room for the everyday negotiation of small, overcrowded, precariously provisioned, conflictive, or non-heteronormative dwellings. As far as migrants are concerned, the complexity of fragmented or multiple housing pathways, composite households, and transnational layers of homing and hospitable obligations makes the everyday negotiation across multiple registers exhausting.

Migrants or refugees are often viewed less as carriers of moral subjectivity and beingness than as a transactional entities to support the host society's survival. Migrants working in manual labour to sustain other people's homes in groceries, factories or agriculture, or others stuck in precarious shelter and legal status are starkly visible in public spaces. The racialized visibility of politically ‘out-of-place’ others serves as a reminder of the provisional, negotiated, and precarious social relations of hospitality and home for strangers. In these circumstances, Goffman's rituals of ‘civil inattention’ to maintain social order are rearticulated in ways that should warrant a minimum of security in encounters with strangers. One does this by recognizing an ‘other's’ presence through eye contact and then averting their gaze, to suggest that one is not a threat – he or she will not ‘invade’ the other's physical space. Migrants demonstrate that hospitality is less a matter of private and domestic concerns than a highly political and public question – whether it is enacted or not, among whom, and on behalf of whom. The spaces and times of hospitality and home through the experience of migrants call for new, theory-driven exploration of its reach and of the emergent space for calibrated reciprocity, along the spatial and temporal lines suggested in this article.

## Notes

1. As anthropologists have long-noted reciprocity is varied and complex. In situations of significant inequality such as refugee and asylum-seeking conditions, these migrants do not have the resources or capacity to return gifts of the same scale as hosts in receiving countries. In that context, forms of social etiquette and expressions serve as provisional ways to acknowledge a social debt – expressions such as gratitude to hosts would be one example. Even here, gratitude is culturally grounded and expressed so can lead to misunderstandings (see Fassin, 2012). There is quite some critical literature in refugee studies on the representation of refugees as the expected behaviour of refugees and conversely those that are ‘ungrateful’ (see Harrell-Bond, 1999; Indra, 1993; Moulin, 2012; Vandevoordt, 2017).

2. Abdoulaye specifically used the language of hospitality to describe how he imagined the obligation one African might feel for another, but we could also see it simultaneously reflecting a sense of solidarity in the face of marginalization and racialization in Italy (thanks to the editor for this observation).
